# Application of a Pharmacogenetics-Based Precision Medicine Model (5SPM) to Psychotic Patients That Presented Poor Response to Neuroleptic Therapy

**DOI:** 10.3390/jpm10040289

**Published:** 2020-12-18

**Authors:** Lorena Carrascal-Laso, Manuel Ángel Franco-Martín, María Belén García-Berrocal, Elena Marcos-Vadillo, Santiago Sánchez-Iglesias, Carolina Lorenzo, Almudena Sánchez-Martín, Ignacio Ramos-Gallego, M Jesús García-Salgado, María Isidoro-García

**Affiliations:** 1 Servicio de Psiquiatría, Hospital Provincial de Zamora, IBSAL, 49071 Zamora, Spain; lorenacarraslaso@gmail.com; 2 Farmacogenética y Medicina de Precisión, Servicio de Bioquímica, Hospital Universitario de Salamanca, IBSAL, 37007 Salamanca, Spain; mbgarcia@saludcastillayleon.es (M.B.G.-B.); elemarcos@hotmail.com (E.M.-V.); mjgarciasal@saludcastillayleon.es (M.J.G.-S.); misidoro@saludcastillayleon.es (M.I.-G.); 3 Servicio de Psiquiatría, Hospital Universitario de Salamanca, IBSAL, 37007 Salamanca, Spain; sisanchez@saludcastillayleon.es (S.S.-I.); carolinalorenzo@usal.es (C.L.); 4 Pharmacogenetics Unit, Pharmacy Department, University Hospital Virgen de las Nieves, UGC Provincial de Farmacia de Granada, Avda, Fuerzas Armadas, 18014 Granada, Spain; almuweb06@gmail.com; 5 Departamento de Fisiología y Farmacología, Universidad de Salamanca, 37007 Salamanca, Spain; ignramos@usal.es; 6 Departamento de Medicina, Universidad de Salamanca, 37007 Salamanca, Spain

**Keywords:** antipsychotic agents, pharmacogenetics, cytochrome P-450 enzyme system, psychotic disorders, precision medicine

## Abstract

Antipsychotics are the keystone of the treatment of severe and prolonged mental disorders. However, there are many risks associated with these drugs and not all patients undergo full therapeutic profit from them. The application of the 5 Step Precision Medicine model(5SPM), based on the analysis of the pharmacogenetic profile of each patient, could be a helpful tool to solve many of the problematics traditionally associated with the neuroleptic treatment. In order to solve this question, a cohort of psychotic patients that showed poor clinical evolution was analyzed. After evaluating the relationship between the prescribed treatment and pharmacogenetic profile of each patient, a great number of pharmacological interactions and pharmacogenetical conflicts were found. After reconsidering the treatment of the conflictive cases, patients showed a substantial reduction on mean daily doses and polytherapy cases, which may cause less risk of adverse effects, greater adherence, and a reduction on economic costs.

## 1. Introduction

The pharmacological tools of the severe mental disorders have been historically scarce until the appearance of the first antipsychotic, which had been used in surgical practice as a sedative agent categorized as an antihistaminic (chlorpromazine) [1]. Its application in psychotic patients was considered a resounding success because of its efficacy on positive symptoms, which led to a drastic reduction in admissions to mental institutions and in the use of more aggressive therapies such as lobotomy or electro convulsive therapy (ECT). These substances were initially defined as “chemical lobotomies” and had a wide repertoire of extrapyramidal effects, among others [2]. 

Second-generation anti-psychotics, entail a lower incidence of extrapyramidal side effects [3,4]. Same way as typical antipsychotics, second-generation antipsychotics bind the dopamine type 2 (D2) receptors, but the latter act selectively on the mesolimbic pathway due to its lower D2 receptor affinity, avoiding extrapyramidal effects when administered in low doses, and have antagonistic effects on the 5-hydroxytryptamine (5-HT) receptors in the cerebral area, which could explain the improvement in negative symptomatology and reduced drug-induced extrapyramidal symptoms [5].

However, empiricism continued to be frequent in clinical practice, with therapeutic decisions based primarily on strategies of trial and error, which prolong the time during which the patient would not receive effective therapy. This practice tends to result in polytherapy, in an effort to cover the entire spectrum of receptors when the search for an efficient monotherapy seems to be unsuccessful [6]. Antipsychotic polytherapy, is not supported by scientific evidence and involves a greater risk of side effects and therapeutic failure [7,8,9,10]. Adverse effects of a specific therapy may cause a special impact on the adherence to the treatment and the therapeutic success [9,10].

Precision Medicine derives from the creation of a diagnostic/prognostic approach, based on genetic, clinical, and environmental information relative to the patient, that makes possible to forecast the response to treatment and opt for the optimal therapeutic option [11,12]. The application of such strategies to routine psychiatric clinical practice may allow solving the problems related to adverse effects that condition the clinical evolution [13,14]. One of the more widespread hypotheses to explain this interindividual variable response to treatment directs the question to a pharmacokinetic cause. Therefore, it is proposed that the metabolism of the neuroleptics administered to the patient could explain these differences [15,16,17].

In our environment, the most commonly used atypical antipsychotics are metabolized mainly through the cytochrome P 450 (CYP450) system [18]. The superfamily CYP450 is a set of highly polymorphic genes, which suggests a possible strong genetic variability among individuals and, consequently, a potential strong phenotypic variability, which translates into different enzymatic activity on the drugs administered to the individual [15,19,20,21,22]. In addition, the therapeutic target of these drugs is found in the central nervous system, meaning they must pass through the blood-brain barrier [23], therefore, the genes involved in the absorption and transport process should be considered [24]. On the other hand, some major side effects related to antipsychotic administration could possibly be related to polymorphism of genes associated with neurotransmitters signaling or catabolic pathways, such as the cathechol-O-methyltransferase (COMT), and the dopamine receptor (DRD) family [25,26]. 

Application of precision medicine can help to decrease adverse events; however, so far it is mostly driven to specific drugs and oriented to genotype of the patient without considering drug-phenoconversion. This is a phenomenon that could cause genotypic extensive metabolizers to behave as phenotypic poor metabolizers [27]. In this sense, we have developed the 5SPM (5 step precision medicine) model [28] that simultaneously evaluates the effects of the pharmacological drug–drug and gene–drug interaction in the complete polypharmacy context of the prescription of each patient. The hypothesis of this study is that the application of 5SPM will lead to a reduction in the dosage of the drug used and in the number of polytherapy cases avoiding adverse effects and therapeutic failure. 

## 2. Materials and Methods

### 2.1. Study Sample

A cohort of 188 patients from the psychiatry units of the Complejo Asistencial de Zamora (Zamora, Spain), Hospital Universitario Rio Hortega (Valladolid, Spain) and Hospital Universitario de Salamanca (Salamanca, Spain), was used for this study, and was analyzed in the Unidad de Medicina de Precisión of the latter. Patients gave informed consent for pharmacogenetic analysis according to the ethics committee of the Hospital Universitario de Salamanca (CEIC ref.: 107/12/2016). Criteria of inclusion were listed as: suffering from a prolonged serious mental illness, poor response to conventional treatment, which eventually results in polypharmacy, with its consequent adverse effects and little support by clinical guides in the matter. Patients who did not receive antipsychotic therapy and were under 16 years of age were excluded. Information was collected on the patient’s diagnosis, current psychopharmacotherapy, and the dose/day used. Likewise, the age, gender, pharmacological history, and significant adverse effects related to antipsychotic therapy or its interactions with concomitant therapy that had been recorded in the patient’s medical history were collected. Clinical and pharmacological data was recorded between 2013 and 2019.

#### Step Precision Medicine

**Figure 1:**
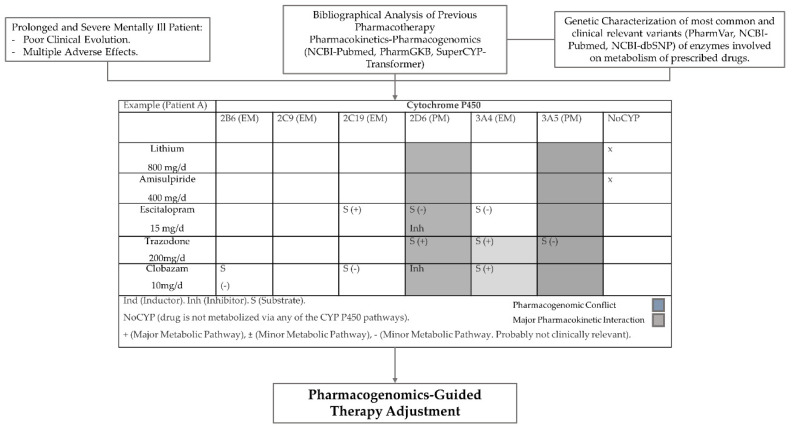
Practical application of the precision medicine model.

The 5SPM method consisting of a five-step precision medicine protocol was applied, including: (1) obtaining of clinical, epidemiological, and therapeutical data, including current prescriptions, diagnosis and therapeutic response; (2) analysis of pharmacological interactions based on the drug-specific pharmacokinetic pathways of drugs included in the study by processing databases such as Pharmacogenomic Knowledgebase (PharmGKB) [29], PubMed-NCBI [30], Charite’s SuperCYP-Transformer [31], and the Pharmacogene Variation (PharmVar) Consortium [32]. The potential drug-–drug and gene drug interaction were analyzed from the previous databases to evaluate the probability of drug-phenoconversion (3) pharmacogenetic analysis of the genes chosen in the study. The genetic markers were selected considering the genes that code enzymes involved in their metabolism (4) correction of pharmacotherapy applied to the patient based on the data obtained by the previous three steps, resolving those cases in which: one or more prescribed drugs were metabolized by an enzyme that had an inefficient phenotype, a pharmacological interaction with influence on the plasma levels of one of the drugs involved was found, or when there was a potential phenoconversion effect due to one or more of the prescribed drugs, fixing those cases in which, there was a detriment to the patient; and (5) the study of results and a reassessment of the model by verifying the evolution of the patients involved (Figure 1, Figure S1). Pharmacogenetic testing (PGx) was performed using the AmpliChip CYP450 Test (Roche Molecular Diagnostics, Pleasanton, CA, USA) [33], the Autogenomics platform, MassARRAY 4.2 (Agena, India), and probe-based assays using the LightCycler platform (Roche Diagnostics, Basel, Switzerland) testing was performed following the directives of the European Molecular Genetics Management Network for DNA handling, with the requisite controls. The application of quality norms followed the UNE-EN-ISO 15189:2007 Normative in the Accredited Section of Molecular Genetics and Pharmacogenetics of the Clinical Biochemistry Service of the University Hospital in Salamanca. The normative included training and qualification of personal, preanalytical, analytical and postanalytical control, blinding, repeating measurements, and internal and external validity.

The genes studied were the ones encoding enzymes 1A2, 2B6, 2C9, 2C19, 2D6, 3A4, 3A5 of the CYP450 cytochrome family. The CYP450 superfamily is composed of highly polymorphic genes (see PharmVar.org). The usual approach to the relationship between genotypic variability and the metabolism of CYP450 substrates is based on the definition of metabolic phenotypes with their characteristic pharmacokinetic implications based on different genetic mechanisms. Poor metabolizers (PM) were associated with two inactive alleles. The combination of two reduced-activity alleles or a reduced-activity with an inactive allele or an inactive allele with an active allele results in an intermediate metabolizer (IM). An individual with two wt-like alleles was labelled as an extensive metabolizer (EM). The presence of a duplication in the absence of inactive or reduced activity alleles results in an ultrarapid metabolizer (UM) [34,35].

Antipsychotic dose comparison was made using chlorpromazine as a reference [36]; chlorpromazine equivalents were calculated by using dose converters to establish a unique chlorpromazine dose value (mg/day) for each patient, before and after the clinical intervention, even if the patient was prescribed with antipsychotic polytherapy.

### 2.2. Statistics

Descriptive statistics were used to determine the central tendency and dispersion. Normality of the distribution was assessed using Kolmogorov–Smirnov test. Statistical power was calculated for sample size using Interactive Statistical Calculation Pages. Wilcoxon Paired Signed Test was used as a paired difference test to determine the difference in distribution of numerical non-gaussian variables before and after the application of PGx testing (i.e., chlorpromazine dose), Mcnemar Test was used to determine the paired homogeneity on nominal data. Statistical power calculation to test the primary hypothesis (reduction of clorpromazine-corrected antipsychotic dose via reduction of each patient’s daily dose and the number of antipsychotic prescribed) was done assuming a mean initial chlorpromazine dose of 600 mg/d with an standard deviation of 1050 mg/d and a minimum clinically important difference of 200 mg/d, obtaining a value greater than 0.80 for an α value of 0.05. 

## 3. Results

### 3.1. Demographics

The results are presented for a total of 188 patients, whose clinical data were collected between 2013 and 2019. The average age of the total study participants was 47.21 (±12.93) years (24–84), with 59.57% of the patients being female (*n* = 112). The distribution in diagnostics is presented in Table 1; the most common diagnosis was paranoid schizophrenia (Diagnostic and Statistical Manual of Mental Disorders, Fifth Edition, DSM-V, F20), which occurred in 67.02% of the cases (*n* = 126). Tobacco smoking was present in 51.38% of the patients (*n* = 93).

### 3.2. Pharmacological Interactions

Data on drug prescribing prior to and after pharmacogenetic analysis are presented in Table 2. A total 343 antipsychotics (Table 2) were prescribed, for an average of 1.82 antipsychotics (range: 1 to 4) per patient, which in the course of this study and after the pharmacogenetic analysis, were reduced to 239, averaging to 1.27 antipsychotics per patient (range 1 to 3), meaning a 30.32% (*p* < 0.05) reduction. 20.75% of patients had more than five drugs prescribed, situation that was reduced to 10.64% (*p* < 0.05) and 71.28% of patients more than one neuroleptic, which goes down to 26.60% (*p* < 0.05) after the 5SPM application. On average, each patient has 14.55 less drugs prescribed and 23.63 less antipsychotics prescribed after the PGx-guided pharmacotherapy adjustment (Table 3) (*p* < 0.05).

**Figure 2:**
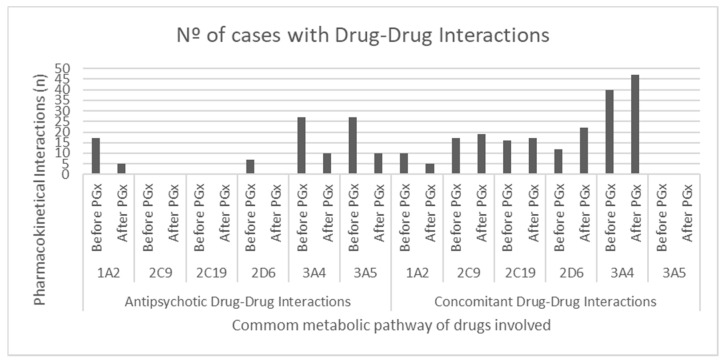
Number of conflicts found associated with each one of the Cytochrome P 450 (CYP450) members studied, before and after pharmacogenetic testing, including neuroleptic and concomitant therapy. PGx: Pharmacogenetic Analysis.

Two or more drugs mainly metabolized by the same enzyme prescribed to the patients were considered as an interaction regardless of the number of drugs involved and was counted independently for each CYP450 member. A total of 173 pharmacological interactions were discovered in the pharmacotherapy of the sample, including neuroleptics and concomitant therapy, both psychiatric and non-psychiatric. The cytochrome through which more conflicts occur was CYP3A4 (*n* = 67), followed by 3A5 (*n* = 27) and 1A2 (*n* = 27). Taking only neuroleptic therapy into account, 78 interactions were tested, most of which occur through cytochromes CYP3A4 (*n* = 27), CYP3A5 (*n* = 27), and CYP1A2 (*n* = 17). Following the modification of pharmacotherapy motivated by the pharmacogenetic study, these interactions were reduced to 135 (CYP3A4, 57; CYP2D6, 22; CYP2C9, 19) and 25 (CYP3A4, 10; CYP3A5, 10; CYP1A2, 5), achieving a reduction in pharmacological interactions of 26.10% (*p* < 0.05) and. 67.95% (*p* < 0.05), respectively (Figure 2). 

For approximately one quarter of the patients receiving polytherapy treatment, it involves the coadministration of olanzapine in infratherapeutic doses for hypnotic purposes, or coadministration of quetiapine for the same purpose. Therefore, there was a small percentage (less than 10%) who truly receives neuroleptic polytherapy as such (Table 4).

### 3.3. Drug-Gene Conflicts

The presence of the alleles listed in Table 5 of the CYP1A2, CYP2B6, CYP2C9, CYP2C19, CYP2D6, CYP3A4, and CYP3A5 genes was analyzed in the Pharmacogenetics and Precision Medicine Unit of the University Hospital of Salamanca. Table 6 shows the frequency of occurrence of the estimated phenotypes from the detection of the different alleles in the sample. Notice that as a Precision Medicine-approach the model is adapted to the specific situation of the patient. Each patient was genotyped, selecting in each case the SNPs that could predict the metabolization of the prescribed therapy. Since each prescription can be different, SNPs can be different between patients. 

The CYP1A2 gene was studied in 179 patients, with the most prevalent phenotype being HI (Higher Inducibility) (86.03%, *n* = 154). Among the 166 patients who were tested for the CYP2B6 gene, the most common phenotype was EM (Extensive Metabolizer) (50.00%, *n*. 83). In reference to the CYP2C9 and CYP2C19 genes (*n* = 183, *n* = 186), the majority phenotype was EM (57.38%, *n* = 105; 47.85%, *n* = 89). Most patients had an EM phenotype (85.95%, *n* = 159; 91.49%, *n* = 172) with respect to CYP2D6 cytochromes (*n* = 183) and CYP3A4 (*n* = 188), and PM (Poor Metabolizer) phenotype (87.85%, *n* = 159) was the most frequent with respect to CYP3A5 cytochrome (*n* = 187).

**Figure 3:**
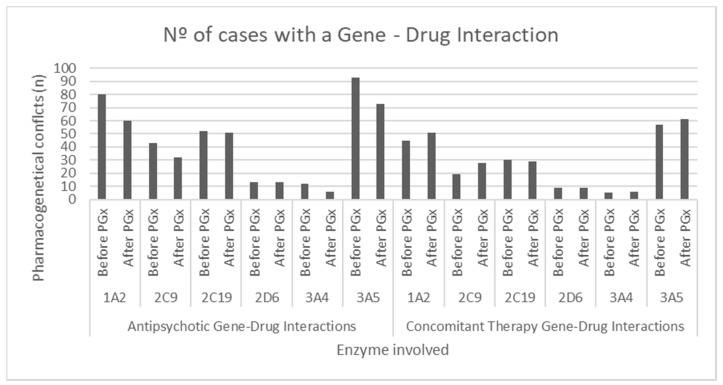
Number of conflicts between the patients’ genotype and the prescribed pharmacotherapy associated with each member of the CYP450 system studied. PGx: Pharmacogenetic Analysis.

In total, taking into account the liver metabolism, mediated through the previously mentioned CYP450 system members, from both antipsychotic therapy (Table 7) [18,31,38] and concomitant therapy, 458 conflicts were discovered between the patient’s prescription and genotype, with most conflicts associated with concomitant therapy occurring through cytochrome CYP3A5 (*n* = 57), and those primarily related to the metabolism of neuroleptics occurring through CYP1A2 (*n* = 80); taking into account the minor metabolizers of the studied antipsychotics, the cytochrome with the highest number of incidences found was CYP3A5 (*n* = 93) (Figure 3). The number of prescriptions of antipsychotics to patients with non-efficient metabolic phenotypes is summarized in Table 8. Following the conduct of the pharmacogenetic study, the total drug-gene conflicts were reduced by 11.02% (*p* < 0.05), based on a reduction in conflicts between neuroleptic therapy and the patient genotype of 19.80% (*p* < 0.05) considering all possible interactions, and 39.02% (*p* < 0.05) considering those that had a noticeable potential impact on the plasma levels of the drug involved. Moreover, with drug-drug interactions, there were more gene-drug interactions associated with non-psychiatric concomitant therapy.

### 3.4. Clinical Impact

The number of prescriptions for the most widely used atypical neuroleptic drugs, together with the prescribed minimum, maximum, and mean dose (mg/day), prior to and after intervention using method 5SPM, are listed in Table 9 and Table 10. It is frequent to see doses beyond the recommended upper limit registered in the pharmacological data sheet of each drug, as frequently prescribe doses were increased by 20–30% before making the decision of considering the pharmacotherapy as a failure and choosing other neuroleptic [39]. As can be seen, the prescription of oral drugs whose metabolism is related to enzymes frequently altered in the study population, is reduced by between 20 and 100%, in favor of intramuscular prescriptions that either do not have liver metabolism, such as Paliperidone, which increases by 186.95%, or whose main route of metabolism is associated with an enzyme that usually has no alterations, such as Aripiprazole, whose prescriptions increase by 100%, predominantly metabolized by CYP3A4. 

**Figure 4:**
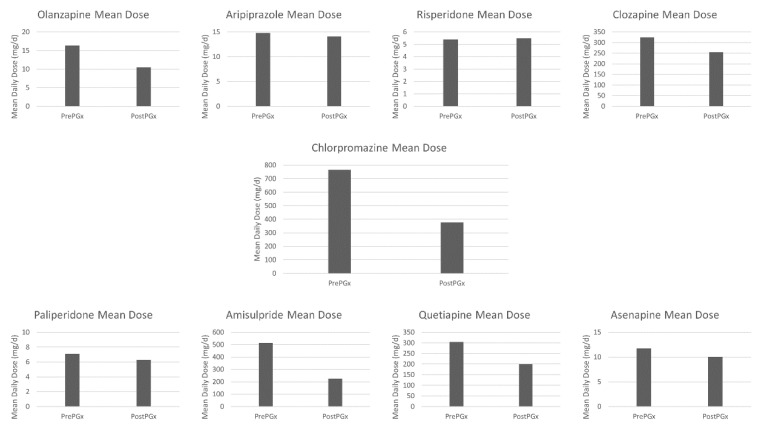
Antipsychotics mean dose and chlorpromazine conversion.

To establish comparisons at the prescribed mean dose of antipsychotic drugs, chlorpromazine was established as a reference drug. The average dose of the different antipsychotics used in the sample per patient, and the average dose of chlorpromazine associated with them, is shown in Figure 4. The reduction by 50.88% (*p* < 0.05) of the mean dose of chlorpromazine per patient in mg/d following the application of the pharmacogenetic study is noteworthy, as an average each patient was prescribed 36.40 less chlorpromazine-corrected antipsychotic dose (384.94 mg/d) (Table 11) (*p* < 0.05). Likewise, the proportion of patients above 800 mg/d was reduced from 34.95% to 1.61% (*p* < 0.05), and the proportion of patients below 300 mg/d increases from 15.05% to 33.87% (*p* < 0.05). A reduction of more than 20% in the dose of chlorpromazine was observed in 78.19% of the sample, achieving a reduction of more than 60% in 23.40% of the sample (*p* < 0.05). 

## 4. Discussion

A patient with prolonged severe mental illness usually has a heterogeneous and highly variable symptomatology, including positive, negative, affective, and cognitive symptomatology, which usually induces the prescriber to opt for the use of the combination of several drugs. However, polypharmacy is not a practice supported by scientific evidence and often carries an increased risk of adverse effects [8], which may imply less adherence to treatment and consequent therapeutic failure, with a higher number of hospital admissions and emergency care, and subsequently increased economic and personal expenditure [19]. 

It is necessary to know the adverse effects profile of neuroleptic treatment in order to perform a risk-benefit balance and thereby facilitate the individualization of the therapy. Polytherapy treatment is considered “justifiable” by some authors given the presence of a profile of adverse effects that hinders adherence to treatment by the patient, or that is detrimental to an underlying pathology of the individual, or when the therapeutic response has not been effective with the antipsychotics available for the specific case of the subject [40]. Consequently, knowledge of the pharmacogenetic profile of the patients becomes more relevant, so that monotherapy adapted to their metabolism can predominantly be applied, suitable for the treatment of symptomatic exacerbations and maintenance, reducing the presence of adverse effects [15]. 

This study is a retrospective descriptive analysis of 188 patients suffering from a prolonged serious mental illness, poor response to conventional treatment, which eventually results in polypharmacy, to whom the 5SPM model was applied. A PGx analysis was carried out aiming to study the main cytochromes involved in both the metabolism of the most commonly used atypical antipsychotics in their clinical centers, as well as in the metabolism of concomitant. It is important to clarify that, even being the application of the 5SPM Model intended as a multidisciplinary effort, the adjustment made on these patients’ pharmacotherapy was mediated by the Psychiatry Department, therefore we discovered more interactions in the sample regarding non-psychiatric concomitanttherapy.

After making corrections on patient pharmacotherapy as part of the 5SPM model, our study revealed a significant reduction in the mean dose of antipsychotic (approximately 50%), which is justified both by the reduction in doses of neuroleptic administered, and by the reduction in approximately 60% of patients receiving two or more antipsychotics.

The pharmacogenetic profile of the population (CYP1A2 HI: 0.86; CYP2D6 EM: 0.85; CYP3A4 EM: 0.91) could explain the need to apply a higher dose with its consequent adverse effects in patients treated with olanzapine [41], clozapine [42], or asenapine [43], all related to CYP1A2, while favoring the use of aripiprazole or risperidone in those who have a wt-like haplotype in CYP2D6 [44,45,46,47,48,49] and CYP3A4 [44,50] cytochromes, a situation frequently found in our population.

The significant decrease in the mean dose of neuroleptic per patient should be discussed, together with the fact of the increase in the prescription of paliperidone and aripiprazole IM (DEPOT), the use of which doubles and almost triples from the beginning of the study. Aripiprazole is a neuroleptic whose metabolism develops predominantly in the liver, through the CYP450 system, specifically by CYP2D6 and CYP3A4 cytochromes which, in the vast majority of our patients (more than 80%), presented an EM phenotype, favoring its use. Paliperidone is an active metabolite of risperidone that has no liver metabolism. This, together with the fact that they are injectable presentations, favors the prevention of side effects and adherence to treatment, with its consequent positive effect when assessing therapeutic success. As mentioned above, there is a decrease in the use of olanzapine (Oral), aripiprazole (Oral), risperidone (Oral and IM), clozapine (Oral), quetiapine (Oral), asenapine (Oral), and paliperidone (Oral) in favor of these long-lasting injectable presentations, presenting better compliance, a potential decrease in hospital admissions, and a reduction in adverse effects. The preference in the use of these antipsychotics is largely determined by the profile of adverse effects associated with them, influenced by the rational approach that this study proposes. In our sample, DEPOT antipsychotics are prescribed frequently as monotherapy and the patients are administered a standardized dose by sanitary personnel, ensuring treatment compliance. Furthermore, gastrointestinal absorption variables, not always totally controlled in this type of patients (such as alcohol consumption), are nullified. One of the main problems of polytherapy is the potential presence of pharmacological interactions, which may lead to an alteration of the biological availability of one of the drugs involved, with the consequent alteration of the therapeutic effect on its target and the presence of possible adverse effects. Drug-induced phenoconversion during routine clinical practice remains a major public health issue (27). This phenomenon needs to be well addressed by precision medicine approaches focused in the drugs in the context of the patient prescription and oriented not only to genotype but considering drug–drug and gene–drug interactions. In fact phenoconversion has been previously called the Achilles’ heel of personalized medicine reporting three principal concerns; drugs susceptible to phenoconversion, co-medications that can cause phenoconversion, and dosage amendments that need to be applied during and following phenoconversion (27). 

The application of 5SPM has helped us to significantly reduce interaction in our patients, either by an increase in the use of drugs not metabolized by the CYP450 system, the decrease in polytherapy previously exposed, or the possible didactic role on medical personnel that the implementation of pharmacogenetic analysis may have provided, regarding the role of the CYP450 system in antipsychotic metabolism. This methodology has led to the discovery of a large number of cases in which there was a problem in the pharmacotherapy due to the metabolism mainly mediated by CYP1A2. In the vast majority of cases, the dose administered per patient has been reduced, as well as cases of polytherapy. It should be noted that the use of DEPOT presentations of drugs such as paliperidone (not metabolized by the CYP450 system) or aripiprazole (whose predominant pathway within the CYP450 system is CYP3A4, which presents a wt-like genotype in the vast majority of the study population) has been the solution for the majority of problematic cases, because of the pharmacogenetic profile of the sample, even considering that each patient was studied individually attending its personal genetic implications. In conclusion, the success achieved in reducing the average dose of antipsychotic administered per patient, which has in turn facilitated the avoidance of a large number of adverse effects and potentially reduced the cost per patient, may be due to the combination of applying a methodology such as 5SPM, focused on the genetic and environmental circumstances of the patient alongside the rise of DEPOT prescriptions, which facilitate adherence to treatment and the use of standardized doses.

## Figures and Tables

**Table 1:** Demographic data.

Variable	Value
PATIENTS	
Total number of patients included:	188
- Average age	47 (±13)
- Male: Female (%)	59.58:37.77
DIAGNOSTIC	
DSM-V *	*n* (%)
F03—Dementia	1 (0.53)
F19—Substance-Related Disorder	12 (6.38)
F20—Schizophrenia	126 (67.02)
F22—Persistent Delusional Disorder	2 (1.06)
F23—Brief and Acute Psychotic Disorder	1 (0.53)
F25—Schizoaffective Disorder	13 (6.92)
F31—Bipolar Disorder	25 (13.30)
F33—Major Depressive Disorder	1 (0.53)
F60—Specific Personality Disorders	2 (1.06)
F61—Mixed Personality Disorder	1 (0.53)
F79—Intellectual Disability	2 (1.06)

* All the pathologies are referred to the official standard nomenclature of the Diagnostic and Statistical Manual of Mental Disorders 5th-edition (DSM-V).

**Table 2:** Antipsychotics prescriptions before/after PGx testing.

Antipsychotic	Presentation	
	Oral (*n* Before/After PGx)	IM (*n* Before/After PGx)
Amisulpride	14/7	
Aripiprazole	38/21	13/26
Asenapine	20/16	
Olanzapine	56/40	
Paliperidone	18/12	23 */66 *
Quetiapine	71/20	
Risperidone	44/2	5/0

PGx: Pharmacogenetic Analysis. * 5 (Before PGx) and 10 (After PGx) patients were prescribed coadjutant oral Paliperidone.

**Table 3:** Drug prescriptions.

Variable	Value
DRUG PRESCRIPTIONS (Pre-PGx Testing/Post-PGx Testing)	
Total Number of drugs (*n*):	635/478
- Average drugs	3.67/3.11
- Average Antipsychotics	1.82/1.27
- More than 5 drugs prescribed (% of total)	20.75/10.64
- More than 1 antipsychotic prescribed (% of total)	71.28/26.60
- Average Within-Patient Drug Variation (%)	−14.55
- Average Within-Patient Antipsychotic Variation (%)	−23.63
Medication classes (% of total)	
A. Digestive system and metabolic	7.24/8.55
B. Blood and hematopoietic organs	0.32/0.00
C. Cardiovascular system	0.95/2.14
D. Dermatologic medications	0.00/0.00
E. Genitourinary apparatus and sex hormones	0.63/0.43
H. Systemic hormone preparations excluded hormones	2.68/4.06
J. Anti-infectious in general for systemic use	0.00/0.00
L. Antineoplastic and immunomodulatory agents	0.16/0.21
M. Skeletal muscle	0.00/0.00
N. Nervous system (Total)	87.72/86.74
N1. Antipsychotics	54.02/51.06
P. antiparasitic products, insecticides, and repellents	0.00/0.00
R. respiratory system	0.00/0.00
S. organs of the senses	0.00/0.00
V. various	0.32/0.00

PGx: Pharmacogenetic Analysis.

**Table 4:** Infratherapeutic dosage percentage.

	Pre-PGx (%)	Post-PGx (%)
Amisulpride (<400 mg)	14	86
Aripiprazole (<10 mg)	16	4
Asenapine (<5 mg)	0	0
Olanzapine (<10 mg)	11	45
Paliperidone (<6 mg)	46	72
Quetiapine (<400 mg)	66	95
Risperidone (<2 mg)	6	0

The criteria to decide what was considered to be the “minimum therapeutic dose” was based on their respective datasheets and the Sthal’s Prescriber Guide. PGx: Pharmacogenetic Analysis.

**Table 5:** Pharmacogenetic testing. Genes and alleles included in the study.

Gene	Alleles
1A2	*1F
2B6	*6
2C9	*1 (WT), *2, *3
2C19	*1 (WT), *2, *4, *17
2D6	*1 (WT), *2, *3, *4, *5, *6, *7, *8, *9, *10, *12, *14, *17, *29, *41, *46
3A4	*1B
3A5	*3C

Alleles listed using the PharmVar Haplotype nomenclature. *: Allele. WT: Wild Type.

**Table 6:** Phenotype profile.

	*n*	PM	IM	EM	UM	HI
CYP1A2	179	-	-	13.97% (*n* = 25)		86.03% (*n* = 154)
CYP2B6	166	9.04% (*n* = 15)	40.96% (*n* = 68)	50% (*n* = 83)	-	
CYP2C9	183	7.10% (*n* = 13)	35.52% (*n* = 65)	57.38% (*n* = 105)	-	
CYP2C19	186	3.23% (*n* = 6)	20.43% (*n* = 38)	47.85% (*n* = 89)	27.96% (*n* = 52)	
CYP2D6	183	3.78% (*n* = 7)	5.95% (*n* = 11)	85.95% (*n* = 159)	3. 24% (*n* = 6)	
CYP3A4	188	1.06% (*n* = 2)	7.45% (*n* = 14)	91.49% (*n* = 172)	-	
CYP3A5	187	87.85% (*n* = 159)	13.81% (*n* = 25)	1.66% (*n* = 3)	-	

Allele Frequencies of the sample were similar to the NCBI dbSNP ALFA Project Frequencies [37]. PM: Poor Metabolizer. IM: Intermediate Metabolizer. EM: Extensive Metabolizer. UM: Ultrarapid Metabolizer. HI: Higher Inducibility.

**Table 7:** Antipsychotic therapy CYP450-mediated metabolism.

	Major Metabolizer	Minor Metabolizer	Product
Olanzapine ^	CYP1A2	CYP2D6 *^,#^	Inactive
Aripiprazole	CYP2D6 ^#,‡^, CYP3A4	CYP3A5 *	Active
Risperidone	CYP2D6 ^#^	CYP3A4 ^#^	Active (Paliperidone)
Amisulpride	NO CYP		
Clozapine	CYP1A2 ^#^, CYP3A4 ^#,‡^	CYP2C19 *^,#^, CYP2C9 *^,#^, CYP2D6 ^#^, CYP3A5 *^,#^	Reduced Activity, Inactive (CYP1A2/CYP3A4)
Paliperidone	NO CYP		
Quetiapine	CYP3A4	CYP3A5 *, CYP2D6 *^,#^	Inactive
Asenapine	CYP1A2	CYP2D6 ^†^	Inactive
Levomepromazine	CYP3A4 ^#^	CYP1A2	Inactive

* Almost null influence on plasma levels, ^#^ substrate inhibition, ^†^ suicide inhibition, ^‡^ inductor, ^^^ inhibits CYP3A4.

**Table 8:** Number of cases (and percentage of the total of individuals presenting each specific phenotype) prescribed with and antipsychotic metabolized by an altered pathway.

*n* before PGx/ *n* after PGx (%)	1A2 HI	2D6 PM	2D6 IM	2D6 UM	3A4 PM	3A4 IM	3A5 PM	3A5 IM
Aripiprazole	-	1 (14.3)/ 1 (14.3)	1 (9.1)/ 2 (18.2)	1 (16.7)/ 0 (0)	1 (50)/0 (0)	7 (50)/ 3 (21.4)	42 (26.4)/ 42 (26.4)	8 (32)/ 5 (20)
Asenapine	11 (7.1)/ 11 (7.1)	1 (14.3)/ 1 (14.3)	2 (18.2)/ 2 (18.2)	0 (0)/0 (0)	-	-	-	-
Clozapine	34 (22.1)/ 21 (13.6)	1 (14.3)/ 2 (28.6)	1 (9.1)/ 1 (9.1)	2 (33.3)/ 1 (16.7)	0 (0)/ 1 (50)	4 (28.6)/ 2 (14.3)	31 (19.5)/ 21 (13.2)	7 (28)/ 4 (16)
Olanzapine	51 (33.1)/ 31 (20.1)	3 (42.9)/ 3 (42.9)	4 (36.4)/ 4 (36.4)	1 (16.7)/ 1 (16.7)	-	-	-	-
Quetiapine	-	2 (28.6)/ 0 (0)	2 (18.2)/ 1 (9.1)	3 (50)/ 1 (16.7)	1 (50)/ 0 (0)	5 (35.7)/ 1 (7.1)	59 (37.1)/ 16 (10.1)	11 (44)/ 4 (16)
Risperidone	-	2 (28.6)/ 0 (0)	2 (18.2)/ 0 (0)	3 (50)/ 0 (0)	0 (0)/ 0 (0)	0 (0)/0 (0)	-	-

HI: Higher Inducibility. PM: Poor Metabolizer. IM: Intermediate Metabolizer. UM: Ultrarapid Metabolizer.

**Table 9:** Mean, Min, and Max Daily Dose prescribed to the study population.

	Pre-PGx			Post-PGx			Variation		
	Mean Dose (mg/d)	Min Dose (mg/d)	Max Dose (mg/d)	Mean Dose (mg/d)	Min Dose (mg/d)	Max Dose (mg/d)	Mean Dose (mg/d)	Min Dose (mg/d)	Max Dose (mg/d)
Olanzapine	16.38	2.5	45	10.5	2.5	40	−0.36	0	−0.11
Aripiprazole	14.79	3	30	14.06	3	30	−0.05	0	0
Risperidone	5.39	1	28.33	5.5	5	6	0.02	4	−0.79
Amisulpride	514.29	100	1000	224.57	100	400	−0.56	0	−0.6
Clozapine	325	100	700	253.85	100	400	−0.22	0	−0.43
Paliperidone	7.08	3	14	5.89	3	9	−0.17	0	−0.36
Quetiapine	304.01	10	1200	199.5	40	600	−0.34	3	−0.5
Asenapine	11.75	5	20	10	5	20	−0.15	0	0

Stahl Prescriber Guide dose ranges (mg/d): amisulpride (400–800), aripiprazole (15–20), asenapine (10–20), clozapine (300–450), olanzapine (10–20), quetiapine (400–800), risperidone (2–8). PGx: Pharmacogenetic Analysis.

**Table 10:** % of prescriptions of the different antipsychotics and presentations.

% Prescriptions	PrePGx	PostPGx	Variation
Olanzapine	29.8	21.3	−28.6
Aripiprazole (Oral)	20.2	11.2	−44.7
Aripiprazole (IM)	6.9	13.8	+100.0
Risperidone	23.4	1.1	−95.5
Risperidone (IM)	2.7	0	−100.0
Clozapine	20.2	13.8	−31.6
Quetiapine	37.8	10.6	−71.8
Asenapine	10.6	8.5	−20.0
Amisulpride	7.4	3.7	−50.0
Paliperidone (Oral)	9.6	6.4	−33.3
Paliperidone (IM)	12.2	35.1	+186.9

**Table 11:** Within-patient variation on antipsychotic daily dose.

	Excluding Therapy Switches		Including Therapy Switches	
Antipsychotic	Mean Variation (%) (CI 95%)	Standard Deviation	Mean Variation (%) (CI 95%)	Standard Deviation
Olanzapine	−30.69 (−35.34, −26.04)	32.54	−30.75 (−42.43, −19.07)	81.71
Aripiprazole	6.37 (−0.24, 12.98)	46.22	−3.22 (−15.19, 8.75)	83.76
Risperidone	0	0	−94 (−98.44, −89.56)	31.05
Amisulpride	−25 (−28.57, −21.43)	25	−39.48 (−51.88, −27.08)	86.73
Clozapine	−23.6 (−27.51, −19.69)	27.33	−27.54 (−39.87, −15.21)	86.24
Paliperidone	−3.7 (−8.32, 0.92)	32.3	40.33 (29.86, 50.79)	73.21
Quetiapine	−24.38 (−30.38, −18.38)	41.95	−73.2 (−81.04, −65.36)	54.87
Asenapine	−13.89 (−16.78, −10.99)	20.23	−16.12 (−28.79, −3.44)	88.67
	Mean Variation (%) (CI 95%)	Standard Deviation		
Chlorpromazine Conversion	−36.4 (−42.12, −30.68)	40.02		

Mean relative within-patient difference in daily dose comparing itself before and after the application of the *5-step Precision Medicine* (5SPM) model. CI: Confidence Interval

## Supplementary Materials

The following are available online at https://www.mdpi.com/2075-4426/10/4/289/s1. Figure S1: 5 Step precision medicine model.
