# Supplementary figures and images for: Application of a Pharmacogenetics-Based Precision Medicine Model (5SPM) to Psychotic Patients That Presented Poor Response to Neuroleptic Therapy
Source: J Pers Med. 2020 Dec 18;10(4):289. doi: 10.3390/jpm10040289 (PMC7767089; doi:10.3390/jpm10040289)

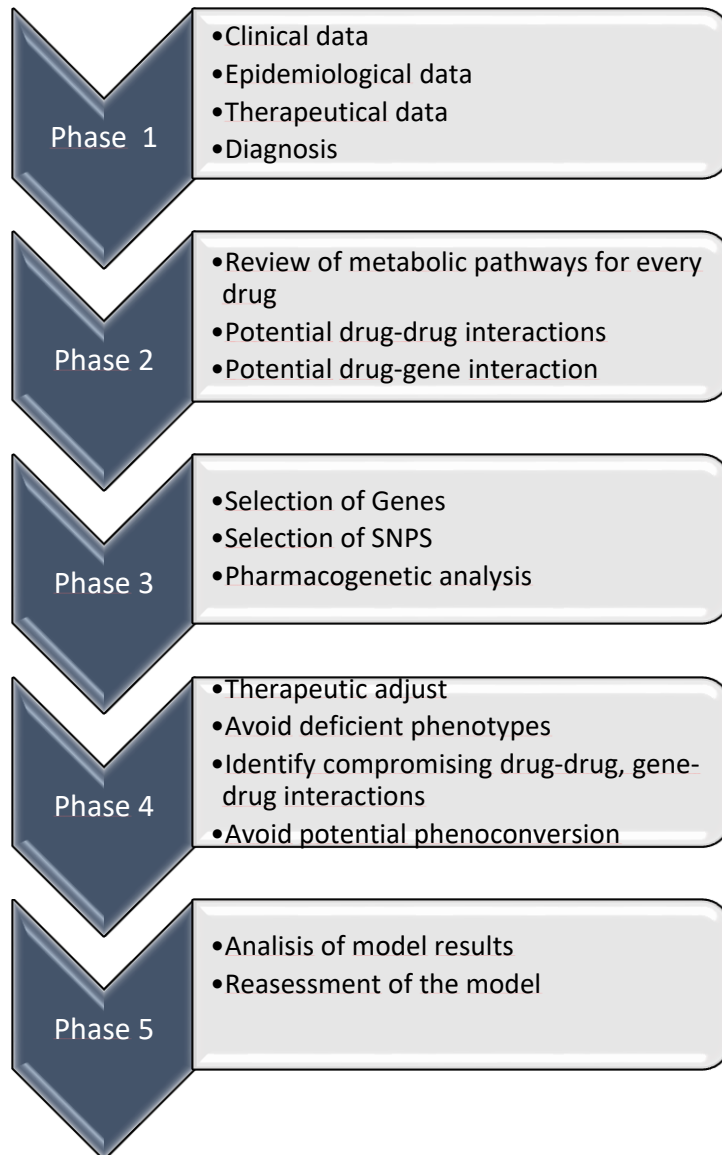

**Suppl. 1.** 5 Step Precision Medicine Model.

Supplement: Supplementary file 1 [file jpm-10-00289-s001.pdf]
